# Combinational therapeutic strategies to overcome resistance to immune checkpoint inhibitors

**DOI:** 10.3389/fimmu.2025.1546717

**Published:** 2025-04-24

**Authors:** Besan H. Alsaafeen, Bassam R. Ali, Eyad Elkord

**Affiliations:** 1 Department of Genetics and Genomics, College of Medicine and Health Sciences, United Arab Emirates University, Al Ain, United Arab Emirates; 2 ASPIRE Precision Medicine Research Institute Abu Dhabi, United Arab Emirates University, Al Ain, United Arab Emirates; 3 Department of Biosciences and Bioinformatics & Suzhou Municipal Key Lab of Biomedical Sciences and Translational Immunology, School of Science, Xi’an Jiaotong-Liverpool University, Suzhou, China; 4 College of Health Sciences, Abu Dhabi University, Abu Dhabi, United Arab Emirates; 5 Biomedical Research Center, School of Science, Engineering and Environment, University of Salford, Manchester, United Kingdom

**Keywords:** immune checkpoint inhibitors, resistance, combination, precision immunotherapy, tumor mircoenvironment

## Abstract

Over the past few years, immune checkpoint inhibitors resulted in magnificent and durable successes in treating cancer; however, only a minority of patients respond favorably to the treatment due to a broad-spectrum of tumor-intrinsic and tumor-extrinsic factors. With the recent insights gained into the mechanisms of resistance, combination treatment strategies to overcome the resistance and enhance the therapeutic potential of immune checkpoint inhibitors are emerging and showing promising results in both pre-clinical and clinical settings. This has been derived through multiple interconnected mechanisms such as enhancing tumor immunogenicity, improving neoantigen processing and presentation in addition to augmenting T cell infiltration and cytotoxic potentials. In the clinical settings, several avenues of combination treatments involving immune checkpoint inhibitors were associated with considerable improvement in the therapeutic outcome in terms of patient’s survival and tumor growth control. This, in turn, increased the spectrum of cancer patients benefiting from the unprecedented and durable effects of immune checkpoint inhibitors leading to their adoption as a first-line treatment for certain cancers. Moreover, the significance of precision medicine in cancer immunotherapy and the unmet demand to develop more personalized predictive biomarkers and treatment strategies are also highlighted in this review.

## 1 Introduction

The past decade has witnessed significant advancements with the use of immune checkpoint inhibitors (ICIs) in treating certain cancers (1). Unlike traditional cancer treatment modalities that function through inducing direct tumor cell death, immunotherapies including ICIs boost the immune system to elicit stronger and more specific anti-tumor immune responses and, therefore, control tumor growth (2). However, despite the unprecedented recent breakthroughs reported with the use of ICIs to treat cancer, not all patients respond similarly to the treatment with only 20-40% showing beneficial outcomes (3, 4). Over the past decade, extensive efforts have been employed to understand the lack of beneficial therapeutic outcome among the treated patients. A growing body of knowledge attributes the resistance to ICIs to various interconnected mechanisms, some of which are tumor-intrinsic while others are external to tumor cells (5). Examples of tumor-intrinsic factors include the lack of neoantigens, impaired tumor antigen processing and presentation, altered oncogenic cell signaling pathways in addition to epigenetic changes that promote drug resistance (5–9). On the other hand, the level of intratumoral immune cell infiltration, the compensatory upregulation of alternate immune checkpoint molecules, angiogenesis and gut microbiome are examples of tumor-extrinsic putative mechanisms that could play a role in inducing resistance to ICIs (5, 10–13).

It is evident that the increased understanding of the resistance mechanisms and underlying factors has -in part- directed the wheel of immunotherapy research toward designing strategies to bypass resistance and enhance the therapeutic potential of ICIs through combining them with other anti-cancer treatment approaches. Despite the limitations associated with conventional cancer treatment modalities, the different adjuvant therapies including chemotherapy, radiotherapy, targeted therapy and epigenetic modulators have shown potentials to shape the tumor microenvironment (TME) by increasing tumor immunogenicity, improving antigen presentation capability of antigen-presenting cells (APCs), enhancing the infiltration of immunoreactive cells to the tumor site and promoting their functional activity (14–16). These changes have been shown to play considerable roles in remodeling the TME and rendering it more conducive to deliver the beneficial effects of ICIs (16). Up to date, considerable number of these strategies demonstrated promising and durable results in terms of efficiency, feasibility and safety, and this has been derived from either the additive or synergistic effects of utilizing ICIs in combination with other therapies (17–19). Some of the combinatorial treatment approaches involving ICIs have already been implemented in the clinical settings to treat specific types of cancer while many others are still at early stages. For instance, the last decade has witnessed remarkable success with the use of chemotherapeutic agents in combination with ICIs to ameliorate resistance and enhance the therapeutic outcome in multiple cancer types. Examples of these treatment modalities that have been approved by the Food and Drug Administration (FDA) include pembrolizumab in combination with carboplatin and either paclitaxel or nab-paclitaxel to treat metastatic squamous non-small cell lung carcinoma (NSCLC), in addition to the combination of nivolumab, cisplatin and gemcitabine that was recently approved to treat metastatic or unresectable urothelial cancer (UC) (20, 21). It is worth appreciating that large number of ongoing pre-clinical and clinical studies are still evaluating the safety and efficiency of combining ICIs with other anti-cancer or immunomodulatory agents in order to expand the spectrum of cancer patients benefiting from immunotherapy. In this review, we discuss multiple strategies to overcome resistance to ICIs from the perspective of combining it with other treatments. Furthermore, the significance of personalized cancer immunotherapy and the need to improve the treatment efficacy in light of precision medicine are also highlighted.

## 2 Potential strategies to overcome resistance by combination therapy

**Figure 1:**
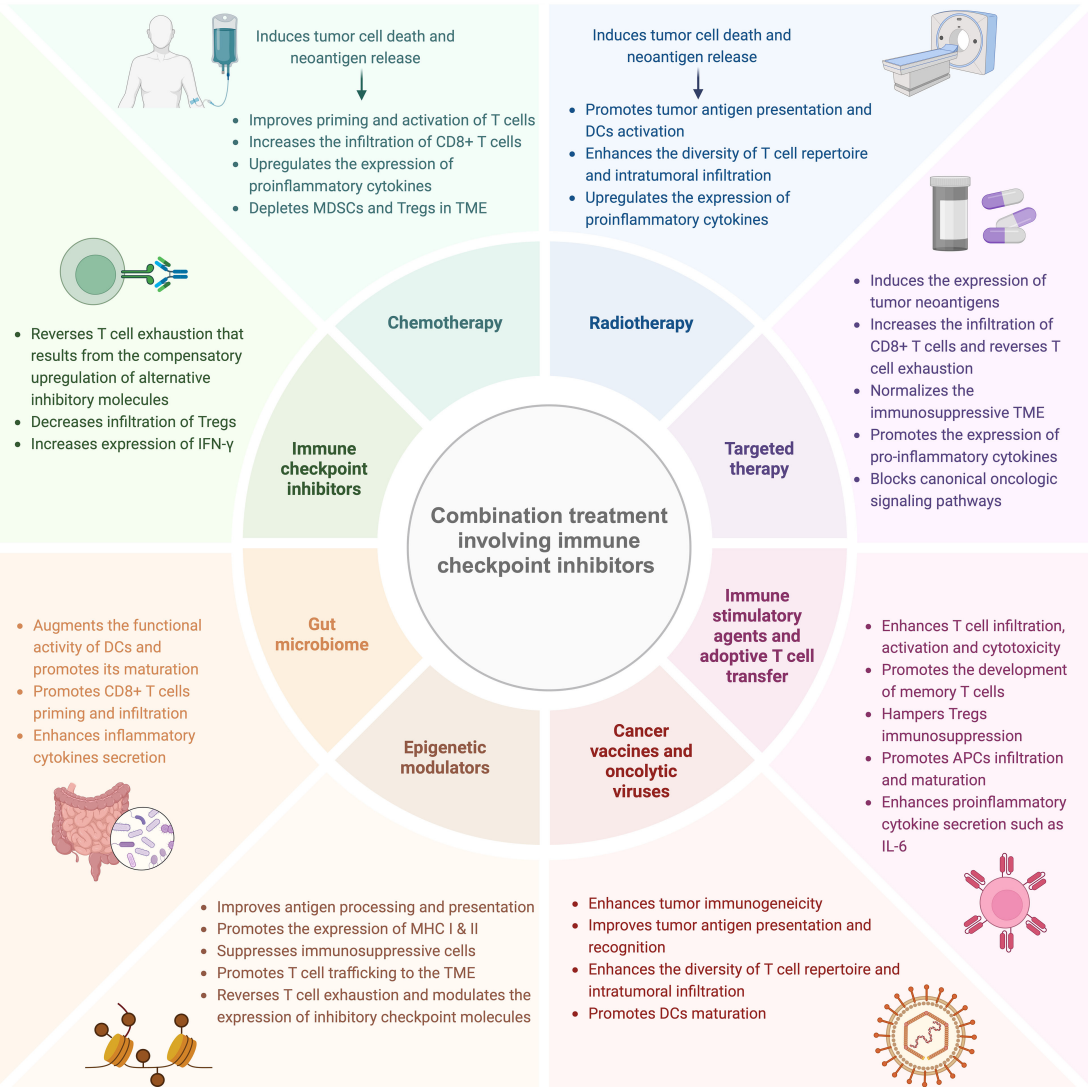
Combination therapeutic strategies to overcome resistance to ICIs and their putative mechanisms of action. Different cancer treatment modalities namely chemotherapy, radiotherapy, dual ICIs, targeted therapy, immune stimulatory agents, epigenetic modulators and gut microbiome manipulation can be employed to ameliorate resistance to ICIs and improve its therapeutic potential through multiple overlapping mechanisms such as enhancing tumor antigenicity, promoting neoantigen processing and presentation in addition to augmenting T cell intratumoral infiltration and functional activity. MDSCs, myeloid-derived suppressor cells; Tregs, regulatory T cells; TME, tumor microenvironment; DCs, dendritic cells; APCs, antigen-presenting cells; MHC, major histocompatibility complex. This figure was created with BioRender.com.

In the last decade, built upon the valuable insights gained into the intrinsic and extrinsic mechanisms of resistance to ICIs, several combination therapeutic approaches were developed in order to overcome resistance and improve the therapeutic efficacy of ICIs. **Figure 1** illustrates the different combination modalities involving ICIs and their putative mechanisms of action.

### 2.1 Combination with chemotherapy

Chemotherapy has been utilized to enhance tumor immunogenicity and overcome resistance to ICIs through inducing tumor cell death and subsequent release of neoantigens (16). This has been shown to improve priming and activation of CD8^+^ cytotoxic T cells (16). In addition, multiple studies have demonestrated the potential of chemotherapeutic agents to remodel the cellular immune system compartment (22–25). For instance, gemcitabine was shown to dramatically and significantly reduce myeloid-derived suppressor cells (MDSCs) in the spleens of tumor-bearing mice while increasing the anti-tumor potential of CD8^+^ T cells and activated natural killer cells (22). Other agents namely cisplatin have been shown to enhance the infiltration of cytotoxic T lymphocytes, upregulate the levels of proinflammatory cytokines as well as the expression of immune checkpoint molecules on intratumoral CD8^+^ T cells (23). Additionally, the role of paclitaxel in modulating the intratumoral immune system has been demonstrated through its capacity to reduce the infiltration of T regulatory cells (Tregs) and stimulate dendritic cells (DC)-mediated antigen presentation (24). Such changes have the potential to alleviate resistance and pave the way to drive the benefits of ICIs among multiple cancer types (26). In a study of advanced melanoma, it was demonstrated that the combination of local chemotherapy (nitrogen mustard alkylating agent melphalan) and anti-CTLA-4 improved response rate and progression-free survival (PFS) through enhancing the infiltration of T cells to the tumor site (27). Other clinical studies of triple-negative breast cancer (TNBC) and NSCLC reported significant improvements in the overall survival (OS) and progression-free survival upon treatment with the combination of chemotherapy (paclitaxel/carboplatin) and ICIs, in comparison to chemotherapy alone (28, 29). In this regard, it is worth noting that not all chemotherapeutic agents are capable to enhance the efficacy of ICIs. For example, vinorelbine and etoposide were not associated with additional benefit when combined with anti-CTLA-4/anti-PD-L1 in murine models of mesothelioma (25). Additionally, the success of chemotherapy and ICIs combination is, in part, dependent on the timing and sequence of chemo immunotherapy administration (30). It is also important to point out that multiple categories of chemotherapy pose different immunomodulatory roles. For instance, fludarabine and cisplatin were shown to interfere with the infiltration and functional capacity of Tregs whereas melphalan and bleomycin were shown to enhance type 1 immunity of Th1 immune response (31, 32). Therefore, it is essential to consider the type and stage of cancer along with the putative resistance mechanism when selecting the chemotherapeutic agent that can be used in combination with ICIs (31, 33). Currently, some combinations of chemotherapy agents and ICIs have become a standard of care to treat some types of cancer in the clinical settings while others are still being investigated in multiple clinical trials (34, 35). A study of NSCLC (KEYNOTE-021) demonstrated that the combination of pembrolizumab (anti-PD1) and chemotherapy (carboplatin and pemetrexed) enhanced the response rate and PFS among treated patients in comparison to those receiving chemotherapy alone (36). This study engaged the FDA to approve the combination of pembrolizumab and standard chemotherapy as a first-line treatment for advanced non-squamous NSCLC (36). Later, other clinical trials examined the potential of combining pembrolizumab and standard chemotherapy to treat squamous NSCLC and documented considerable improvement in patient’s OS and PFS, compared to chemotherapy (37, 38). The results of these studies led to the FDA approval of pembrolizumab in combinationwith chemotherapy for the treatment of NSCLC patients. Based on a string of success along with the remarkable improvement in the therapeutic outcome, the indications of chemotherapy in combination with ICIs were extended to include a wider spectrum of cancer patients, for example, the combination of atezolizumab (anti-PD-L1) and chemotherapy was approved to treat metastatic non-squamous NSCLC (atezolizumab plus nab-paclitaxel and carboplatin), unresectable locally advanced or metastatic TNBC (atezolizumab plus nab-paclitaxel) and extensive-stage small cell lung cancer (atezolizumab plus carboplatin and etoposide) (39–41). **Table 1** provides a summary for the FDA-approved chemoimmunotherapeutic regimens and their indications.

**Table 1:** List of FDA-approved combination immunotherapies that involve ICIs as of July 2024.

Combination	Drug	FDA approved indication	Date of approval	REF
Combination with chemotherapy	Keytruda (pembrolizumab) in combination with pemetrexed and carboplatin	Metastatic non-squamous non-small cell lung carcinoma (NSCLC)	May 10, 2017	(42)
	Keytruda (pembrolizumab) combined with pemetrexed and platinum	Metastatic non-squamous NSCLC without EGFR or ALK genomic tumor aberrations	Aug 20, 2018	(43)
	Keytruda (pembrolizumab) combined with carboplatin and either paclitaxel or nab-paclitaxel	Metastatic squamous NSCLC	Oct 30, 2018	(20)
	Tecentriq (atezolizumab) combined with bevacizumab, paclitaxel and carboplatin	Metastatic non-squamous NSCLC without EGFR or ALK genomic tumor aberrations	Dec 6, 2018	(44)
	Tecentriq (atezolizumab) plus Abraxane (nab-paclitaxel)	Metastatic triple-negative or unresectable locally advanced breast cancer (TNBC) in people with PD-L1 positive tumors	Mar 8, 2019	(39)
	Tecentriq (atezolizumab) combined with carboplatin and etoposide	Extensive-stage small cell lung cancer	Mar 18, 2019	(41)
	Keytruda (pembrolizumab), platinum and fluorouracil (FU) combined	Metastatic or with unresectable, recurrent head and neck squamous cell carcinoma (HNSCC)	June 11, 2019	(45)
	Tecentriq (atezolizumab) combined with Abraxane (nab-paclitaxel) and carboplatin	Metastatic non-squamous NSCLC without EGFR or ALK genomic tumor aberrations	Dec 3, 2019	(40)
	Opdivo (nivolumab) plus Yervoy (ipilimumab) given with two cycles of platinum-doublet chemotherapy	Metastatic or recurrent NSCLC without EGFR or ALK genomic tumor aberrations.	May 26, 2020	(46)
	Keytruda (pembrolizumab) combined with paclitaxel, nab-paclitaxel or gemcitabine and carboplatin	Locally recurrent unresectable or metastatic TNBC whose tumors are PD-L1 positive	Nov 13, 2020	(47)
	Keytruda (pembrolizumab) in combination with platinum- and fluoropyrimidine-based chemotherapy	Locally advanced or metastatic esophageal or gastroesophageal junction (GEJ) carcinoma that is not amenable to surgical resection or definitive chemoradiation	Mar 23, 2021	(48)
	Opdivo (nivolumab) combined with fluoropyrimidine- and platinum-containing chemotherapy	Advanced or metastatic gastric cancer, GEJ cancer, and esophageal adenocarcinoma	April 16, 2021	(49)
	Keytruda (pembrolizumab),trastuzumab, fluoropyrimidine- and platinum-containing chemotherapy combined	Locally advanced unresectable or metastatic HER2-positive gastric or GEJ adenocarcinoma	May 5, 2021	(50)
	Keytruda (pembrolizumab) in combination with chemotherapy and then continued as a single agent after surgery	Early-stage TNBC	July 27, 2021	(51)
	Keytruda (pembrolizumab) combined with chemotherapy with or without bevacizumab	Persistent, recurrent or metastatic cervical cancer whose tumors that express PD-L1	Oct 13, 2021	(52)
	Opdivo (nivolumab) combined with platinum-doublet chemotherapy	Resectable NSCLC	Mar 4, 2022	(53)
	Opdivo (nivolumab) combined with fluoropyrimidine- and platinum-containing chemotherapy	Metastatic or unresectable advanced esophageal squamous cell carcinoma (ESCC)	May 27, 2022	(54)
	Imfinzi (durvalumab) in combination with gemcitabine and cisplatin	Metastatic or locally advanced biliary tract cancer	Sep 05, 2022	(55)
	Libtayo^®^ (cemiplimab) combined with platinum-based chemotherapy	Advanced NSCLC without EGFR, ALK or ROS1 aberrations	Nov 8, 2022	(56)
	*Imfinzi* (durvalumab) combined with *Imjudo *(tremelimumab) and platinum-based chemotherapy	Stage IV (metastatic) NSCLC	Nov 11, 2022	(57)
	Jemperli (dostarlimab) in combination with carboplatin and paclitaxel, followed by Jemperli as a single agent	Primary advanced or recurrent endometrial cancer that is mismatch repair deficient (dMMR)	July 31, 2023	(58)
	Keytruda (pembrolizumab) combined with platinum-containing chemotherapy as neoadjuvant treatment followed by pembrolizumab as a single agent after surgery	Resectable NSCLC	Oct 16, 2023	(59)
	Keytruda (pembrolizumab) in combination with gemcitabine and cisplatin	Metastatic or locally advanced unresectable biliary tract cancer	Nov 1, 2023	(60)
	Keytruda (pembrolizumab) combined with fluoropyrimidine- and platinum-containing chemotherapy	Locally advanced unresectable or metastatic gastric or GEJ adenocarcinoma that are human epidermal growth factor receptor 2 (HER2)-negative	Nov 16, 2023	(61)
	Padcev with Keytruda (pembrolizumab)	Locally advanced or metastatic urothelial cancer (UC)	Dec 15, 2023	(62)
	Opdivo (nivolumab) in combination with cisplatin and gemcitabine	Unresectable or metastatic UC	Mar 7, 2024	(21)
	Imfinzi (durvalumab) in combination with carboplatin and paclitaxel, and then continued as a single agent	dMMR primary advanced or recurrent endometrial cancer	June 14, 2024	(63)
	Keytruda (pembrolizumab) in combination with carboplatin and paclitaxel, and then continued as a single agent	Primary advanced or recurrent endometrial carcinoma	June 17, 2024	(64)
Combination with chemoradiotherapy	Keytruda (pembrolizumab) in combination with chemoradiotherapy	Stage III-IVA cervical cancer	Jan 12, 2024	(65)
Combination with ICIs	Opdivo (nivolumab) in combination with Yervoy (ipilimumab)	BRAF V600 wild-type unresectable or metastatic melanoma	Oct 1, 2015	(66)
	Opdivo (nivolumab) combined with Yervoy (ipilimumab)	BRAF V600 wild-type and mutation-positive unresectable or metastatic melanoma	Jan 23, 2016	(67)
	Opdivo (nivolumab) in combination with Yervoy (ipilimumab)	Intermediate and poor-risk advanced RCC	April 16, 2018	(68)
	Opdivo (nivolumab) in combination with Yervoy (ipilimumab)	Microsatellite instability (MSI) high dMMR metastatic colorectal cancer (CRC) that has showed progression after treatment with fluoropyrimidine, oxaliplatin and irinotecan	July 11, 2018	(69)
	Opdivo (nivolumab) plus Yervoy (ipilimumab)	HCC patients who have been previously treated with sorafenib	Mar 11, 2020	(70)
	Opdivo (nivolumab) plus Yervoy (ipilimumab)	Metastatic NSCLC whose tumors express PD-L1	May 15, 2020	(71)
	Opdivo (nivolumab) plus Yervoy (ipilimumab)	Unresectable malignant pleural mesothelioma	Oct 2, 2020	(72)
	Opdualag (combination of nivolumab and relatlimab)	Unresectable or metastatic melanoma	Mar 18, 2022	(73)
	Opdivo (nivolumab) plus Yervoy (ipilimumab)	Unresectable advanced or metastatic ESCC	May 27, 2022	(54)
	Imjudo (tremelimumab) in combination with Imfinzi (durvalumab)	Unresectable hepatocellular carcinoma (HCC)	Oct 24, 2022
(74)	Combination with targeted therapy	Keytruda (pembrolizumab) in combination with Inlyta (axitinib)	Advanced RCC	April 22, 2019	(75)
Bavencio (avelumab) in combination with Inlyta (axitinib)		Advanced RCC	May 14, 2019	(76)
Keytruda (pembrolizumab) plus Lenvima		Advanced endometrial carcinoma that is not MSI-high or and demonstrated disease progression following prior systemic therapy and are not eligible for surgery or radiation	Sep 17, 2019	(77)
Tecentriq (atezolizumab) in combination with Avastin (bevacizumab)		Unresectable or metastatic HCC who did not receive prior systemic therapy	May 29, 2020	(78)
Tecentriq (atezolizumab) plus Cotellic (cobimetinib) and Zelboraf (vemurafenib) combined		Advanced melanoma patients with BRAF V600 mutation	July 30, 2020	(79)
Opdivo (nivolumab) combined with Cabometyx (cabozantinib)		Advanced RCC	Jan 22, 2021	(80)
Keytruda (pembrolizumab) plus Lenvima		Advanced endometrial carcinoma that is not MSI-high or dMMR, who have disease progression following prior systemic therapy and are not eligible for surgery or radiation therapy	July 22, 2021	(81)
Keytruda (pembrolizumab) plus Lenvima		Advanced RCC	Aug 11, 2021	(82)
Keytruda (pembrolizumab) combined with Padcev (enfortumab vedotin-ejfv)		Locally advanced or metastatic UC who are not candidates for cisplatin-containing chemotherapy	April 3, 2023	(83)

NSCLC, non-small cell lung cancer; TNBC, triple-negative breast cancer; HNSCC, head and neck squamous cell carcinoma; GEJ, gastroesophageal junction; ESCC, esophageal squamous cell carcinoma; HCC, hepatocellular carcinoma; dMMR, mismatch repair deficient; UC, urothelial cancer; RCC, renal cell carcinoma; MSI, microsatellite instability; CRC, colorectal cancer.

### 2.2 Combination with radiotherapy

Radiotherapy is thought to enhance tumor sensitivity to ICIs through inducing tumor cell death and neoantigen release. This, in turn, enhances the diversity of T cell receptor (TCR) repertoire of tumor-infiltrating lymphocytes and promote tumor antigen presentation (14). Other studies have demonstrated that radiotherapy enhances the secretion of proinflammatory cytokines and the activation of DCs, leading to increased accumulation of intratumoral lymphocytes (16). In a study of melanoma, it was reported that the combination of local radiation therapy and anti-CTLA-4 immunotherapy enhanced the therapeutic outcome in comparison to anti-CTLA-4 monotherapy by enhancing the infiltration of CD8^+^ T cells (84). In line with this study, a combination of radiotherapy, anti-PD-L1 and anti-TIM-3 has been shown to inhibit tumor growth, improve survival, enhance the cytotoxic activity of T cells and decrease infiltration of Tregs in a murine model of head and neck squamous cell carcinoma (HNSCC) (85). Despite this favorable outcome, durable responses were not observed due to the resurgence of Tregs (85). A recent study of metastatic NSCLS reported that the combination of radiotherapy and ICIs was associated with improved outcome only in patients who were PD-L1 negative (86). Moreover, it is worth noting that the total dose, fractionation mode of radiotherapy and sequence of radiotherapy and ICIs administration have impact on modulating the immune response and efficacy of ICIs (86–88). Overall, it is appreciated that radiation therapy plays a role in transforming the TME from non-immunogenic to immunogenic and, therefore, enhance the sensitivity to ICIs (87).

### 2.3 Combination with targeted therapy

Several lines of evidence documented the potential of different approaches of targeted therapy such as angiogenesis inhibitors, EGFR inhibitors, HER2 inhibitors and hormone receptor inhibitors to overcome the resistance associated with ICIs (17). This was shown to be mediated through different mechanisms that increase tumor sensitivity to immunotherapeutic agents. For example, targeting mitogen-activated protein kinase (MAPK) pathway utilizing EGFR/MEK/BRAF inhibitors was shown to induce the expression of tumor neoantigens, promote the infiltration of CD8^+^ T cells to the tumor site and block the canonical oncologic signaling pathways (16). In line with these findings, multiple pre-clinical and clinical trials demonstrated that the combination of these inhibitors and PD-1/PD-L1 blockade resulted in improved anti-tumor immunity and overall therapeutic outcomes (89–92). In addition, pre-clinical studies utilizing PI3K inhibitors in combination with ICIs documented the combination’s potential to inhibit tumor growth and improve overall survival (93, 94). This outcome was associated with increased expression of pro-inflammatory cytokines and enhanced T cell cytotoxicity. Moreover, several pre-clinical and clinical studies demonstrated the capacity of other approaches of molecular targeted therapy namely vascular endothelial growth factor (VEGF) inhibitors, indoleamine 2,3-dioxygenase (IDO) inhibitors, A2A receptor blockade and others to reverse the resistance to ICIs through multiple mechanisms such as normalizing the immunosuppressive TME, enhancing the infiltration of intratumoral T cells in addition to reversing T cell exhaustion and promoting its effector function (15–17, 95). For instance, targeting VEGF, an immunosuppressive factor, has been shown to augment endothelial cell activation, inhibit tumor neovascularization and reinforce the anti-cancer immunity (96, 97). Another study demonstrated that bevacizumab treatment, a VEGF inhibitor, resulted in trends towards increase in the expression of genes associated with effector CD8^+^ T cells, T-helper chemokines and natural killer cells (98). Overall, these changes were shown to enhance tumor sensitivity to ICIs in which the combination treatment was associated with superior outcomes in comparison to monotherapy. Additionally, therapies targeting adenosine axis such as anti-CD38 and anti-CD73 inhibitors succeeded in overcoming resistance associated with ICIs through their potential to increase the infiltration of intratumoral effector CD4^+^ T cells, enhance the expression of granzyme B and IFN-γ by tumor-infiltrating CD8^+^ T cells and decrease the accumulation of the immunosuppressive MDSCs and Tregs within the TME (99, 100).

### 2.4 Combination with other immune checkpoint inhibitors

Given that the compensatory upregulation of alternative inhibitory immune checkpoint molecules is one of the challenges to achieve favorable outcome with ICIs, multiple pre-clinical and clinical trials were directed to utilize multiple blockade as a putative mechanism to bypass resistance (101). The combination of anti-CTLA-4 and anti-PD-1 was associated with improved therapeutic outcomes in perspective to patient’s survival (102). The clinical benefit derived from ICIs combination treatment could be attributed to the complimentary mechanisms in which anti-CTLA-4 functions through promoting T cell priming while anti-PD-1 plays a key role in enhancing the effector function of T cells. Moreover, a recent study in melanoma patients revealed that anti-CTLA-4 induces a robust clonal expansion of progenitor exhausted T cells which, in turn, promotes exhausted T cell reinvigoration when combined with anti-PD-1 treatment (103). Such combination has been approved to treat multiple cancer types including melanoma, hepatocellular carcinoma, renal cell carcinoma and colorectal cancer (104–106). Additionally, various pre-clinical trials have demonstrated the synergistic beneficial outcome of targeting PD-1/PD-L1 axis and other inhibitory immune checkpoint molecules including TIM-3, LAG-3, VISTA, BTLA and TIGIT in terms of tumor growth control and overall survival (107–112). This has been shown to be associated with reversed T cell exhaustion, increased expression of IFN-γ and decreased levels of Tregs. Besides the FDA approved dual ICIs treatment (**Table 1**), multiple other combinations are under evaluation by a significant number of clinical trials (113–115).

### 2.5 Combination with immune stimulatory agents

It is well-appreciated that the magnitude of T cell activation and subsequent anti-tumor immune responses is determined by the dynamic interplay between co-stimulatory and inhibitory immune checkpoint molecules. One way to improve T cell activation is through targeting immune stimulatory pathways, and therefore, provide a rationale for immune stimulatory agonist including CD40, ICOS, OX40, GITR and 41BB to be utilized in combination with ICIs to treat cancer patients. Several pre-clinical and clinical trials documented the potential of these immune stimulatory agonists to alter the intratumoral immune system compartment and promote the beneficial response to ICIs by enhancing T cell infiltration, activation and cytotoxicity (15, 16). In addition, other studies reported the capacity of the immune stimulatory ICOS agonist and GITR agonist to improve the therapeutic outcome through promoting the development of memory T cells and hampering Treg immunosuppression, respectively (116, 117). Moreover, the role of other immunostimulatory agents including CD40 agonist to increase infiltration of DCs, reduce MDSCs and promote APC maturation has been documented (118, 119). This, in turn, may contribute to its potential to overcome ICIs resistance.

### 2.6 Combination with adoptive T cell transfer

Adoptive T cell transfer has led to considerable clinical benefits in treating hematological malignancies; however, this success was limited in solid tumors due to poor infiltration of T cells along with the immunosuppression imposed by the TME. Multiple pre-clinical studies demonstrated that the combination of ICIs with adoptive T cell transfer improved therapeutic outcome in terms of tumor regression and improved survival (120, 121). This was thought to be delivered through enhancing the intratumoral infiltration of T cells as well as the secretion of proinflammatory cytokines such as IL-6. The first-in-human clinical trial that utilized adoptive T cell transfer in combination with ICIs demonstrated that treating melanoma patients with a combination of ipilimumab and adoptive cell transfer is feasible and well-tolerated (122). Another clinical study reported that engineered CD19 chimeric antigen receptor (CAR)-T cells expressing IL-7 and CCL19 in combination with anti-PD-1 improved the anti-tumor immune response and long-term remission rate in relapsed or refractory diffuse large B cell lymphoma patients (123). It is worth noting that IL-7 and CCL9 play a considerable role in promoting the proliferation, infiltration, accumulation and survival of CAR-T cells in lymphoid tissues (124, 125). This, in turn, was shown to be associated with enhanced anti-tumor potentials and improved therapeutic outcome compared to conventional CAR-T cells (125–127). Overall, it can be concluded that the capacity of adoptive T cell transfer to augment the recruitment and functional activity of T cells could act synergistically with ICIs in order to enhance treatment efficacy (121, 122, 128).

### 2.7 Combination with cancer vaccines and oncolytic viruses

It is known that lack of neoantigens is one of the mechanisms that confer resistance to immunotherapy. Therefore, tumor vaccines have been utilized in order to enhance tumor immunogenicity, augment anti-tumor immune responses, and pave the way to overcome resistance and drive the clinical benefits of ICIs. A significant number of pre-clinical and clinical studies provided a strong evidence that cancer vaccines can significantly improve the therapeutic response to ICIs in multiple cancer types (129–131). This was shown to be mediated by the capacity of cancer vaccines to (a) improve antigen presentation and recognition, (b) expand the repertoire of neoantigen-specific T cells and (c) enhance intratumoral infiltration of T cells (16, 129, 130). Moreover, the first-in-human clinical trials illustrated the safety, feasibility and immunotherapeutic potential of targeting tumor mutations (130–132). In a study of melanoma, it was demonstrated that the combination of nivolumab (anti-PD-1) and tumor neoantigen vaccines was associated with complete tumor regression and enhanced patient’s survival (130, 131). Additionally, durable and complete tumor regression was also documented in metastatic cancer patients following treatment with a combination of neoantigen-loaded monocyte-derived dendritic cell vaccine and anti-PD-1 (133). Given that patients with the same tumor type have distinct mutational signature, the use of personalized cancer vaccines is thought to enhance treatment specificity and effectiveness. On the other hand, cancer vaccines require HLA haplotype compatibility and could result in off-target immune stimulation (134). This, in turn, may limit its application in the clinical use.

In a similar manner to cancer vaccines, oncolytic viruses showed the potential to enhance the therapeutic outcome of ICIs through promoting antigen presentation and T cell priming. The combination of ICIs namely ipilimumab or pembrolizumab and talimogene laherparepvec- a genetically modified oncolytic herpes simplex virus 1 that express granulocyte-macrophage colony-stimulating factor (GM-CSF)-demonstrated improved therapeutic potential in treating unresectable melanoma patients in comparison to monotherapy without any additional safety concerns (135–139). The virus was designed to replicate selectively in cancerous cells and induce lytic cell death and release of tumor-specific antigens (136). This, in turn, was proven to enhance the anti-tumor immune response through promoting DC maturation, IFN-γ expression, T cell activation and infiltration (136). In fact Talimogene laherparepvec is the first oncolytic viral immunotherapy to be approved by the FDA to treat locally advanced or unresectable melanoma patients. Nowadays, multiple pre-clinical and clinical trials are underway in the effort of testing the efficacy of combining ICIs with Talimogene laherparepvec and other novel oncolytic viruses (140).

### 2.8 Combination with epigenetic modulators

It is known that epigenetic dysregulation plays a crucial role in cancer development and progression. Therefore, targeting this axis has been associated with promising results in multiple malignancies. Beyond their potential as monotherapies, several lines of evidence documented the considerable role of epigenetic modulators such as DNA methyltransferase inhibitors and histone deacetylase inhibitors (HDACi) to improve the sensitivity of cancerous cells to chemotherapy, radiotherapy, targeted therapy and some approaches of immunotherapy (141). The improved efficacy of ICIs when combined with epigenetic modulators was shown to be derived from the potential of combination treatment to modulate the TME (142–146). Multiple studies have demonstrated the capacity of Entinostat- a selective class I HDACi- to enhance the therapeutic potential of PD-1 blockade through inhibiting the immunosuppressive function of MDSCs, suppressing Tregs, enhancing MHC class I expression in addition to promoting the infiltration and functional activity of intratumoral T cells (142–145). Another study of advanced NSCLC showed that a pan HDACi namely Vorinostat is capable to prime the TME and enhance the response to PD-1 inhibitors through enhancing the expression of immune-related genes, inhibiting immunosuppressive cells as well as promoting the production of pro-inflammatory cytokines (146). Moreover, it was reported that DNA methyltransferase inhibitors are capable to reverse the epigenetic silencing of T helper 1 immunostimulatory chemokines including CXCL9 and CXCL10 and, in turn, promote T cell trafficking to the TME and enhance the sensitivity to immunotherapeutic agents such as ICIs and adoptive T cell transfer (9). In a pre-clinical study of colorectal cancer, it was demonstrated that HBI-8000 – a HDACi- augmented the treatment outcome in synergy with CTLA4/PD-1/PD-L1 blockade and resulted in improved tumor growth control (147). This was shown to be mediated by the capacity of HBI to alter the TME from being immunologically cold (nonresponsive) to becoming hot (responsive) through modulating the expression of inhibitory immune checkpoints, enhancing the functional activity of APCs and modulating the expression of MHC I and II (147). Additionally, another pre-clinical study of colorectal and metastatic mammary cancers illustrated that both DNA methyltransferase inhibitors and HDACi are capable to improve the response to CTLA-4 and PD-L1 inhibitors through targeting the immunosuppressive MDSCs (148). Currently, a number of clinical studies are undergoing to assess the translation of these promising results from pre-clinical to clinical settings in perspective to safety and efficacy (149–153).

### 2.9 Combination with gut microbiome

Given that the composition of gut microbiota plays remarkable roles in shaping the anti-tumor immunity, modulating the microbiome was associated with augmented efficacy of ICIs as demonstrated by multiple pre-clinical and clinical studies. Using a pre-clinical model of melanoma, Sivan et al. demonstrated that the oral administration of Bifidobacterium abolished tumor outgrowth when combined with PD-L1 inhibitors (154). This synergistic effect was driven by the capacity of the combination treatment to augment DC function, promote CD8^+^ T cell priming and infiltration, in addition to enhancing inflammatory cytokines secretion (154). In line with these findings, it has been reported that the oral administration of *Bacteroides fragilis* with *Burkholderia cepacian* or *Bacteroides thetaiotaomicron* enhanced the efficacy of anti-CTLA-4 through promoting DC maturation and T cell-mediated immune responses (155). Another study demonstrated the improved tumor growth control accompanied with enhanced T cell response and improved efficiency of PD-L1 blockade following reconstitution of germ-free mice with fecal material from patients responding to ICIs (156). Moreover, gut microbiome manipulation was shown to abrogate immune-related adverse events that are associated with ICIs treatment. Wang et al. demonstrated that the oral administration of *Lactobacillus reuteri* provided a protective effect and inhibited the development of colitis through decreasing the distribution of group 3 innate lymphoid cells that are induced by ICIs-related colitis (157). Such findings have set the stage for clinical trials aiming to assess the safety, feasibility and efficacy of modulating gut microbiome to enhance the therapeutic potential of ICIs among cancer patients (155, 158, 159). Recent clinical trials elucidated insights into the potential of utilizing fecal microbiota transplantation to induce favorable changes in the intratumoral immune cell compartment and gene expression profiles in gut and tumor tissues of patients with PD-1 inhibitors-refractory metastatic melanoma and these were translated into promising clinical benefits (158, 159).

### 2.10 Other strategies to improve the efficacy of ICIs

Multiple other strategies combining ICIs with other approaches are being investigated with some demonstrating promising results. For example, several lines of evidence documented the potential of cytokines namely IL-2, IL-12 and IL-15 in synergy with ICIs to improve the overall therapeutic outcome (160–162). This was shown to be associated with enhanced effector T cell and natural killer cell responses. Additionally, targeting chemokines plays a considerable role in modulating the TME in favor of tumor inhibition. For instance, utilizing CCR-1/2 antagonists and CXCR-1/2 inhibitors was proven to reduce MDSC infiltration in the TME and inhibit epithelial-mesenchymal transition (EMT) (163–166). Such changes were associated with favorable responses to ICIs in multiple cancer types. Moreover, immunosuppressive cells including Tregs, MDSCs and tumor-associated macrophages (TAMs) could be targeted to bypass resistance to ICIs using different agents such as anti-CCR-4 inhibitors, agonistic TRAIL-R antibody and CSF-1R inhibitor (15, 16). These modulators in synergy with ICIs have shown potential to reverse the immunosuppressive nature of the TME and suppress tumor invasion, metastasis and angiogenesis (16). In addition, some studies demonstrated that bacterial-mediated cancer therapy has the potential to augment the antitumor immune response and ameliorate resistance to ICIs (167–169). Al-Saafeen et al. demonstrated the capacity of attenuated *Salmonella typhimurium* to improve the efficacy of anti-PD-L1 in a pre-clinical model of colorectal cancer through enhancing intratumoral T cell infiltration, upregulating the expression of MHC II and decreasing the percentage of tumor-associated granulocytic cells (168). Several other studies have also demonestrated the potential of certain bacterial strains and their metabolites to remarkably influence the therapeutic outcome of ICIs through modulating the TME and promoting T cell activation (155, 156, 170–173). In one study using epithelial tumors, Routy et al. showed that *Akkermansia muciniphila* is capable to enhance DCs maturation, IL-2 secretion and T cell infiltration leading to a better response to anti-PD-1 immunotherapy (171). On the other hand, some bacterial genera, namely Ruminococcus and Roseburia, have been associated with poor response to ICIs due to their role in inducing immunosuppressive cells (170). Despite promising results, implementing this approach in the clinical settings still presents some challenges regarding dosing, safety and other factors. Overall, the favorable outcome associated with the combination treatments is thought to be based on the potential of adjuvant therapy to modulate the TME and transform it from being immunosuppressive to becoming immunogenic and, therefore, pave the way for ICIs to deliver its beneficial therapeutic effect. This comes in addition to utilizing combination treatments in order to target different tumorigenesis promoting axes and abrogate immune evasion fostering a synergistic beneficial outcome. To this point, several pre-clinical and clinical studies are underway in the effort of enhancing the therapeutic potential of ICIs and expanding the spectrum of responsive patients through identifying the mechanisms of resistance and targeting it by combining ICIs with other approaches.

## 3 Precision cancer immunotherapy

The marked variation in the response to immunotherapy among cancer patients along with the tumor complexity and heterogeneity paved the way for personalized medicine and highlighted its significance for cancer immunotherapy. Precision medicine is a promising approach in oncology that utilizes the intra- and intertumoral genetic and epigenetic variability, the tumor immune microenvironment, in addition to patient’s lifestyle and the surrounding environmental factors to provide the best-fit treatment and prevention strategies for the patient (174). In other words, precision medicine utilizes the comprehensive genomic, transcriptomic and proteomic data to guide treatment choices. Through identifying specific genetic mutations and alterations in signaling pathways along with characterizing the intratumoral immune system compartment, this approach facilitates tailoring the therapy to the unique characteristics of each patient in order to enhance the treatment efficacy while keeping the associated adverse events to the minimal (175). This would expand the fraction of patients who can clinically benefit from ICIs.

Patient’s response to ICIs is a complex trait shaped by several intrinsic and extrinsic factors. Nowadays, three FDA-approved predictive biomarkers are used for routine patient’s selection and those are (a) PD-L1 expression, (b) Microsatellite instability (MSI) and (c) tumor mutational burden (TMB) (176–178). Given that each of these biomarkers has certain limitations and utilizes different assays for various cancer types, there is a lack of a standardized, well-defined framework for implementing these biomarkers in patient selection criteria (178). Consistently, data from 18,792 patients across 100 peer-reviewed studies revealed that patients predicted to respond beneficially to ICIs- based on FDA-approved biomarkers- showed only a small degree of overlap suggesting that each of these biomarkers contribute differently to the overall response (179). Moreover, the use of a single biomarker isn’t feasible to predict the response to immunotherapeutic agents (180–183). Some biomarker-negative patients have shown favorable response to the treatment while other patients with similar levels of these biomarkers showed variable responses to the treatment. For example, the expression of PD-L1 has been associated with beneficial responses to PD-1/PD-L1 blockade across multiple cancer types; however, other studies demonstrated that patients with PD-L1 negative tumors can still achieve favorable outcomes when treated with PD-1/PD-L1 inhibitors (184–187). It is worth noting that the intratumoral heterogeneity and dynamic nature of PD-L1 expression across the tumor-tissue could influence the reliability of PD-L1 as a predicative biomarker for the response to anti-PD-1/PD-L1 immunotherapy (188). Additionally, neoantigen load, immune infiltration status and IFN-γ signaling pathway genes were shown to exhibit heterogeneity among discrete regions within the same tumor in a single patient (189). Therefore, tumor sampling might not precisely reflect the distribution of these parameters which, in turn, compromise their predictive value. In addition, multiple studies have demonstrated the association of high TMB and corresponding neoantigen load with the enhanced sensitivity to ICIs whereas others showed that certain mutations played a role in shaping the overall response to ICIs independently from the tumor mutational load (190–194). Therefore, the limited predictive values of these FDA-approved biomarkers stressed the unmet need to identify highly accurate and more personalized predictive biomarkers to assist patient’s selection in clinical settings. New class of predictive biomarkers related to gene-based expression signature has emerged- though not yet approved by the FDA- including T cell-inflamed gene expression profile (195), T cell dysfunction and exclusion gene signature (196), melanocytic plasticity signature (197, 198), and B cell-focused gene signature (198, 199). These biomarkers were shown to have superior and enhanced predictive value compared to single gene or protein markers. Moreover, recent studies have highlighted the use of differentially expressed non-coding RNA (ncRNA) to predict the response to immunotherapy (200). In a study of NSCLC, specific circulating miRNA namely miR-93, -138-5p, -200, -27a and -424 were shown to be remarkably highly expressed in patients who demonstrated favorable response to anti-PD-1 compared to non-responding patients (201). This emphasizes on the significance of utilizing ncRNAs as crucial biomarkers for the early response to ICIs (201). In line with this, it has been demonstrated that the immune functional long ncRNAs signature is associated with favorable response to ICIs, superior tumor growth control, augmented intratumoral infiltration of cytotoxic T cells and PD-L1 expression (202). An additional study of TNBC documented the association between high levels of long ncRNA LINK-A and resistance to PD-1 blockade (203). This has been attributed to the potential of LINK-A expression to enhance the degradation of the antigen peptide-loading complex and the intrinsic tumor suppressors *Rb* and *p53* interfering with tumor antigenicity and tumor’s intrinsic suppression capacity (203). Overall, it is worth appreciating that the utilization of ncRNAs in patient’s selection would substantially improve the capacity to predict the immunotherapeutic clinical outcome. Over the past decade, multiple molecular tools including PCR-based tests, conventional sequencing, RNA-sequencing and next-generation sequencing were utilized for patient’s enrollment in the clinical trials of targeted cancer therapies and have been approved by the FDA as standard procedures to guide and select the appropriate treatment for cancer patients (175, 204). These technologies help in providing a comprehensive cancer genomic landscape and identifying predictive and prognostic molecular alterations that could result in the resistance to a specific therapeutic agent. However, the efficacy of these methods can be associated with some limitations. For instance, whole exome-sequencing- used in most of the studies- identifies genomic associations with the therapeutic response in the exome regions that represent approximately 1% of the whole genome. However, it does not capture variations in the non-coding regions, which may have predictive value for treatment response or be associated with specific mechanisms of resistance (205, 206). Moreover, the application of these technologies in the clinical settings is still limited due to tumor heterogeneity and the intratumoral variation that could interfere with responsiveness to molecular-targeted therapies (204). It is evident that tumor cells during cancer development acquire genetic mutations and undergo changes in the epigenetic signature resulting in different populations of tumor cells that vary in their morphology, genotype and function (207). The selective pressure allows cancerous cells that acquired mutations with survival and therapy-resistance advantages to dominate tumor tissue. Recent studies assumed that the source of tumor heterogeneity is a population of undifferentiated malignant cells with stem-like phenotype (208, 209). These cells were capable of self-renewal and differentiation into different tumor cells in response to the TME (210). Therefore, more advanced technologies are needed to overcome this obstacle and provide more efficient and personalized cancer treatment.

## 4 Conclusions and future perspectives

The increased understanding of the resistance mechanisms facilitated efforts to overcome the resistance through targeting the underlying mechanisms using combination treatment strategies. Several lines of evidence demonstrated the potential of combination treatments to reverse the resistance by leveraging the synergistic effects of targeting different immune evasion mechanisms, resulting in improved therapeutic outcome and overall response to the treatment. In addition to combinatorial treatment modalities, the response rate and therapeutic efficacy of ICIs can be enhanced through implementing accurate personalized treatment. Several predicative biomarkers from the tumor, peripheral blood and other sites have been identified to improve patient’s selection for treatment. However, clinical studies have shown that the prognostic value of a single biomarker isn’t sufficient to predict the response to the treatment taking into account that the clinical predictive values of the identified biomarkers don’t apply to all treated patients. These findings paved the path for the personalization of cancer immunotherapy and tailoring the treatment strategy for individual patients. Nowadays, extensive efforts to fine-tune personalized predictive biomarkers using advanced technologies are ongoing. For example, single-cell RNA-sequencing (scRNA-seq) is considered a powerful high-throughput transcriptomics method that uncover tumor complexity and dynamics by profiling the gene expression of each single cell within the tumor tissue (210). It also enables a more refined understanding of the mechanistic details of the multifactorial and dynamic interactions that occur in the TME, in addition to providing insights into the specific immunogenic markers associated with the heterogenous cancerous clones. Overall, scRNA-seq has shown a great promise in improving the efficiency and application of precision cancer immunotherapy. Despite the substantial ongoing efforts, there is an increasing demand to design a multi-omics approach that integrates DNA, RNA, proteomic and metabolomic analyses to provide a better understanding of the tumor biology, tumor tissue heterogeneity, the mechanistic details of the anti-tumor immune responses, in addition to the key drivers of inter-patient variability in response to immunotherapy. Such approach will help in identifying innovative predictive biomarkers, designing novel targeted therapies and optimizing treatment strategies to include combinatorial therapies. This, in turn, should play a remarkable role in ameliorating the resistance to immunotherapeutic agents and improve the overall response rate.
