# Correction: Combinational therapeutic strategies to overcome resistance to immune checkpoint inhibitors

**DOI:** 10.3389/fimmu.2026.1831008

**Published:** 2026-04-24

**Authors:** Besan H. Alsaafeen, Bassam R. Ali, Eyad Elkord

**Affiliations:** 1 Department of Genetics and Genomics, College of Medicine and Health Sciences, United Arab Emirates University, Al Ain, United Arab Emirates; 2 ASPIRE Precision Medicine Research Institute Abu Dhabi, United Arab Emirates University, Al Ain, United Arab Emirates; 3 Department of Biosciences and Bioinformatics & Suzhou Municipal Key Lab of Biomedical Sciences and Translational Immunology, School of Science, Xi’an Jiaotong-Liverpool University, Suzhou, China; 4 College of Health Sciences, Abu Dhabi University, Abu Dhabi, United Arab Emirates; 5 Biomedical Research Center, School of Science, Engineering and Environment, University of Salford, Manchester, United Kingdom

**Keywords:** immune checkpoint inhibitors, resistance, combination, precision immunotherapy, tumor mircoenvironment

There was an error in **Table 1** as originally published. Some FDA-approved immune checkpoint inhibitor (ICI) combination therapies were unintentionally omitted and certain entries required updating to accurately reflect the FDA approvals as of July 2024. The corrected **Table 1**, along with the updated references, now provides a comprehensive list of FDA-approved combination immunotherapies involving ICIs as of July 2024.

**Table 1:** List of FDA-approved combination immunotherapies that involve ICIs as of July 2024.

Combination	Drug	FDA approved indication	Date of approval	REF
Combination with chemotherapy	Keytruda (pembrolizumab) in combination with pemetrexed and carboplatin	Metastatic non-squamous non-small cell lung carcinoma (NSCLC)	May 10, 2017	(1)
	Keytruda (pembrolizumab) combined with pemetrexed and platinum	Metastatic non-squamous NSCLC without EGFR or ALK genomic tumor aberrations	Aug 20, 2018	(2)
	Keytruda (pembrolizumab) combined with carboplatin and either paclitaxel or nab-paclitaxel	Metastatic squamous NSCLC	Oct 30, 2018	(3)
	Tecentriq (atezolizumab) combined with bevacizumab, paclitaxel and carboplatin	Metastatic non-squamous NSCLC without EGFR or ALK genomic tumor aberrations	Dec 6, 2018	(4)
	Tecentriq (atezolizumab) plus Abraxane (nab-paclitaxel)	Metastatic triple-negative or unresectable locally advanced breast cancer (TNBC) in people with PD-L1 positive tumors	Mar 8, 2019	(5)
	Tecentriq (atezolizumab) combined with carboplatin and etoposide	Extensive-stage small cell lung cancer	Mar 18, 2019	(6)
	Keytruda (pembrolizumab), platinum and fluorouracil (FU) combined	Metastatic or with unresectable, recurrent head and neck squamous cell carcinoma (HNSCC)	June 11, 2019	(7)
	Tecentriq (atezolizumab) combined with Abraxane (nab-paclitaxel) and carboplatin	Metastatic non-squamous NSCLC without EGFR or ALK genomic tumor aberrations	Dec 3, 2019	(8)
	Opdivo (nivolumab) plus Yervoy (ipilimumab) given with two cycles of platinum-doublet chemotherapy	Metastatic or recurrent NSCLC without EGFR or ALK genomic tumor aberrations.	May 26, 2020	(9)
	Keytruda (pembrolizumab) combined with paclitaxel, nab-paclitaxel or gemcitabine and carboplatin	Locally recurrent unresectable or metastatic TNBC whose tumors are PD-L1 positive	Nov 13, 2020	(10)
	Keytruda (pembrolizumab) in combination with platinum- and fluoropyrimidine-based chemotherapy	Locally advanced or metastatic esophageal or gastroesophageal junction (GEJ) carcinoma that is not amenable to surgical resection or definitive chemoradiation	Mar 23, 2021	(11)
	Opdivo (nivolumab) combined with fluoropyrimidine- and platinum-containing chemotherapy	Advanced or metastatic gastric cancer, GEJ cancer, and esophageal adenocarcinoma	April 16, 2021	(12)
	Keytruda (pembrolizumab),trastuzumab, fluoropyrimidine- and platinum-containing chemotherapy combined	Locally advanced unresectable or metastatic HER2-positive gastric or GEJ adenocarcinoma	May 5, 2021	(13)
	Keytruda (pembrolizumab) in combination with chemotherapy and then continued as a single agent after surgery	Early-stage TNBC	July 27, 2021	(14)
	Keytruda (pembrolizumab) combined with chemotherapy with or without bevacizumab	Persistent, recurrent or metastatic cervical cancer whose tumors that express PD-L1	Oct 13, 2021	(15)
	Opdivo (nivolumab) combined with platinum-doublet chemotherapy	Resectable NSCLC	Mar 4, 2022	(16)
	Opdivo (nivolumab) combined with fluoropyrimidine- and platinum-containing chemotherapy	Metastatic or unresectable advanced esophageal squamous cell carcinoma (ESCC)	May 27, 2022	(17)
	Imfinzi (durvalumab) in combination with gemcitabine and cisplatin	Metastatic or locally advanced biliary tract cancer	Sep 05, 2022	(18)
	Libtayo^®^ (cemiplimab) combined with platinum-based chemotherapy	Advanced NSCLC without EGFR, ALK or ROS1 aberrations	Nov 8, 2022	(19)
	*Imfinzi* (durvalumab) combined with *Imjudo* (tremelimumab) and platinum-based chemotherapy	Stage IV (metastatic) NSCLC	Nov 11, 2022	(20)
	Jemperli (dostarlimab) in combination with carboplatin and paclitaxel, followed by Jemperli as a single agent	Primary advanced or recurrent endometrial cancer that is mismatch repair deficient (dMMR)	July 31, 2023	(21)
	Keytruda (pembrolizumab) combined with platinum-containing chemotherapy as neoadjuvant treatment followed by pembrolizumab as a single agent after surgery	Resectable NSCLC	Oct 16, 2023	(22)
	Keytruda (pembrolizumab) in combination with gemcitabine and cisplatin	Metastatic or locally advanced unresectable biliary tract cancer	Nov 1, 2023	(23)
	Keytruda (pembrolizumab) combined with fluoropyrimidine- and platinum-containing chemotherapy	Locally advanced unresectable or metastatic gastric or GEJ adenocarcinoma that are human epidermal growth factor receptor 2 (HER2)-negative	Nov 16, 2023	(24)
	Padcev with Keytruda (pembrolizumab)	Locally advanced or metastatic urothelial cancer (UC)	Dec 15, 2023	(25)
	Opdivo (nivolumab) in combination with cisplatin and gemcitabine	Unresectable or metastatic UC	Mar 7, 2024	(26)
	Imfinzi (durvalumab) in combination with carboplatin and paclitaxel, and then continued as a single agent	dMMR primary advanced or recurrent endometrial cancer	June 14, 2024	(27)
	Keytruda (pembrolizumab) in combination with carboplatin and paclitaxel, and then continued as a single agent	Primary advanced or recurrent endometrial carcinoma	June 17, 2024	(28)
Combination with chemoradiotherapy	Keytruda (pembrolizumab) in combination with chemoradiotherapy	Stage III-IVA cervical cancer	Jan 12, 2024	(29)
Combination with ICIs	Opdivo (nivolumab) in combination with Yervoy (ipilimumab)	BRAF V600 wild-type unresectable or metastatic melanoma	Oct 1, 2015	(30)
	Opdivo (nivolumab) combined with Yervoy (ipilimumab)	BRAF V600 wild-type and mutation-positive unresectable or metastatic melanoma	Jan 23, 2016	(31)
	Opdivo (nivolumab) in combination with Yervoy (ipilimumab)	Intermediate and poor-risk advanced RCC	April 16, 2018	(32)
	Opdivo (nivolumab) in combination with Yervoy (ipilimumab)	Microsatellite instability (MSI) high dMMR metastatic colorectal cancer (CRC) that has showed progression after treatment with fluoropyrimidine, oxaliplatin and irinotecan	July 11, 2018	(33)
	Opdivo (nivolumab) plus Yervoy (ipilimumab)	HCC patients who have been previously treated with sorafenib	Mar 11, 2020	(34)
	Opdivo (nivolumab) plus Yervoy (ipilimumab)	Metastatic NSCLC whose tumors express PD-L1	May 15, 2020	(35)
	Opdivo (nivolumab) plus Yervoy (ipilimumab)	Unresectable malignant pleural mesothelioma	Oct 2, 2020	(36)
	Opdualag (combination of nivolumab and relatlimab)	Unresectable or metastatic melanoma	Mar 18, 2022	(37)
	Opdivo (nivolumab) plus Yervoy (ipilimumab)	Unresectable advanced or metastatic ESCC	May 27, 2022	(17)
	Imjudo (tremelimumab) in combination with Imfinzi (durvalumab)	Unresectable hepatocellular carcinoma (HCC)	Oct 24, 2022	(38)
Combination with targeted therapy	Keytruda (pembrolizumab) in combination with Inlyta (axitinib)	Advanced RCC	April 22, 2019	(39)
	Bavencio (avelumab) in combination with Inlyta (axitinib)	Advanced RCC	May 14, 2019	(40)
	Keytruda (pembrolizumab) plus Lenvima	Advanced endometrial carcinoma that is not MSI-high or and demonstrated disease progression following prior systemic therapy and are not eligible for surgery or radiation	Sep 17, 2019	(41)
	Tecentriq (atezolizumab) in combination with Avastin (bevacizumab)	Unresectable or metastatic HCC who did not receive prior systemic therapy	May 29, 2020	(42)
	Tecentriq (atezolizumab) plus Cotellic (cobimetinib) and Zelboraf (vemurafenib) combined	Advanced melanoma patients with BRAF V600 mutation	July 30, 2020	(43)
	Opdivo (nivolumab) combined with Cabometyx (cabozantinib)	Advanced RCC	Jan 22, 2021	(44)
	Keytruda (pembrolizumab) plus Lenvima	Advanced endometrial carcinoma that is not MSI-high or dMMR, who have disease progression following prior systemic therapy and are not eligible for surgery or radiation therapy	July 22, 2021	(45)
	Keytruda (pembrolizumab) plus Lenvima	Advanced RCC	Aug 11, 2021	(46)
	Keytruda (pembrolizumab) combined with Padcev (enfortumab vedotin-ejfv)	Locally advanced or metastatic UC who are not candidates for cisplatin-containing chemotherapy	April 3, 2023	(47)

NSCLC, non-small cell lung cancer; TNBC, triple-negative breast cancer; HNSCC, head and neck squamous cell carcinoma; GEJ, gastroesophageal junction; ESCC, esophageal squamous cell carcinoma; HCC, hepatocellular carcinoma; dMMR, mismatch repair deficient; UC, urothelial cancer; RCC, renal cell carcinoma; MSI, microsatellite instability; CRC, colorectal cancer.

Following the table revision, the sentence describing the timing of FDA approval for the combination of pembrolizumab and chemotherapy in squamous NSCLC could be misleading. This correction has been made to ensure consistency with the updated table.

A correction has been made to Section 1.1 “*Combination with chemotherapy*”:

Original sentence:

“In 2018, the results of these studies have led to the FDA approval of pembrolizumab and chemotherapy for the treatment of squamous NSCLC patients (37, 38).”

Corrected sentence:

“The results of these studies led to the FDA approval of pembrolizumab in combination with chemotherapy for the treatment of NSCLC patients.”
